# Observations on Cholera

**Published:** 1854-09

**Authors:** 


### HALL'S JOURNAL OF HEALTH.

VOL. I.] SEPTEMBER, 1854. [NO. IX.

### OBSERVATIONS ON CHOLERA.

In the August number, I have insisted mainly on

1st. An *uncomfortableness about the belly* as the very earliest premonition of approaching Cholera, in cholera times.

2d. That at this stage, an almost infallible and immediate cure is effected by prompt and perfect quietude on the back, on a bed, satisfying the thirst, if any, by swallowing pellets of ice, and eating, only if decidedly hungry, farinaceous food, tea and toast, or thickened gruel; and that this course should be continued until the feeling in the abdomen has entirely disappeared, and until there is a desire to walk about, and a sensation of pleasure or relief in doing so.

3d. That if in cholera times, there has been no passage from the bowels in two or three days, or if there be three passages from the bowels in any twenty-four hours, or a single passage of a watery and light-colored substance, or an unaccountable feeling of weakness, amounting almost to prostration, without any noticed looseness, or constipation, or nausea, or abdominal uncomfortableness, in either of these four conditions, most especially the last, a resident physician, in whom high confidence is reposed, should be at once consulted.

4th. That if the symptoms are urgent, such as two or three lightish-colored, painless, watery passages, in the course of five or six hours, or vomiting or cramps, and a physician cannot be had in the course of three or four hours, *then*, in addition to the quietude on the back, a flannel bandage firmly fastened around the abdomen, and eating ice, if there is thirst, as a precaution, and to be on the safe side, and to save time which may be infinitely valuable to the patient, a calomel pill of ten grains should at once be swallowed; and if the vomiting or purging do not cease within two hours, and a physician does not arrive, then swallow *two* of the calomel pills.

If the patient is afraid of being salivated, then let him take twice as much super carbonate of Soda as he has taken calomel, in pills, or dissolved in a tablespoon or two of cold or warm water. It is not necessary that the calomel should be in the form of a pill; if there is no vomiting or decided nausea, the next best method of taking it is to put it on the end of a spoon-handle or case-knife, put it in the mouth, and, suddenly turning it over, spread or plaster the calomel on the back part of the tongue, and wash it down with ice-water. Then chew afterwards any tough substance, such as a piece of dried beef, or tough bread crust, so as to clean the teeth and mouth from any particles of calomel which may have obtained a lodgment —and, even after that, rinse the mouth out well, otherwise the teeth may be injured. The prejudices against calomel have arisen from its indiscriminate and careless use. In precisely the same manner have prejudices quite as strong arisen against the use of tea and coffee, and roast beef, and fruits, until our whole dietetic table is reduced to grapes and cold water.

Intelligent men have written against the use of calomel in cholera; but in every case I have lately seen reported, as proof of the inefficacy of calomel, one of two things invariably attended that case—either *other things* were done or given with the calomel, such as opium, or salts, or ipecac, or jalap, or rhubarb,—or the patient died in spite of all subsequent treatment, bringing us back to the admitted point, that where calomel fails all other things will fail. All that I have said in reference to the good effects of calomel in cholera, is to be considered as applicable to cases where nothing else has been *given* but pure calomel—where nothing else has been *done* but lying on the back on a bed, and eating ice, if thirsty. When calomel does not arrest the watery passages, it is because enough is not given; or it is a fatal case. Since writing the Cholera article, an intelligent gentleman connected with one of our oldest and most respectable publishing houses in Broadway, has informed me that a medical gentleman in the eastern part of the city has made a large amount of money at five dollars a case, and that, from his success, his whole time is fully occupied. His main treatment is from twenty to forty grains of calomel at the first dose, and bathing the feet in hot water saturated with the salt of a fish barrel.

I have said nothing about the subsequent or convalescing treatment of cholera, diet, &c., as it is a disease so critically dangerous that it is madness not to secure the services of a regular practising physician, even when the treatment advised has been followed with the happiest results.

I wish it to be distinctly understood, that in the calomel treatment, everything else taken or done beside the ice and quiet, is a positive injury, unless under the direction of a physician; for any prescription however familiar—and these are the things which we denominate "*simple, and can do no harm, even if they do no good*"—even a mustard plaster over the stomach or abdomen may excite an irritation in the system difficult to control; and sometimes, as I have seen, it produces unutterable torture: a patient once begged with dying earnestness to have it removed, if it were "but for five minutes."

Another "*simple*" is *paregoric*, a household medicine, the common destroyer of the health and lives of young children in the hands of ignorant mothers and lazy, unprincipled nurses. Ten, twenty, fifty drops of paregoric have been so often given under various circumstances, that it, too, is so familiar as to have become one of the *simples*, and it does faithfully act towards arresting the passages, and life too, by convulsions, apoplexies or fatal congestions. A grain of opium, twenty drops of laudanum, or a teaspoon of paregoric,—either one is capable of causing convulsions immediately, when they act so as to arrest the looseness, suddenly.

It is the use of opiates in loose bowels which explains the fact that among the eleven hundred and thirty-nine deaths in New York city, for the last reported week in July of this year, five hundred and thirty-three were from bowel affections, and one hundred and seventy-nine, besides, from congestions of various kinds,—opiates acting uniformly in one of two ways, soothing the disease for the moment, to break out with greater aggravation in a short time; or, on the other hand, to act in a more summary manner, causing congestions and more sudden death.

The startling fact forces itself on our attention, that now, in August, 1854, every other death in New York is from disorder of the bowels, bringing us back to the point, that the very slightest bowel affection in cholera times, demands instantaneous attention. One week later: total deaths, 1148; congestions, 133; disease of the bowels, 645—more than one-half.

In the week ending July 22d, there were nine hundred and fifteen deaths, four hundred and twelve of which were from diseases of the bowels, and ninety-seven more of convulsions and congestions. One of our most estimable citizens recently died with a short sickness, reported of cholera, but his three attending physicians certified through the papers that "he died of congestive fever." If this distinguished gentleman had loose bowels at first, as the papers stated, and took anodynes in any form to arrest the looseness, then it was death from cholera, badly treated; and the statement that he died of "congestive fever" is not full, and misleads. Let my readers remember whenever they see a death recorded from convulsions, apoplexy, or congestion in any form, in cholera times, that such a death, in nine cases out of ten, has followed some anodyne or high stimulant taken into the stomach. I have no objection to the use of an injection of two or three teaspoonfuls of laudanum in as many tablespoons of water, or introducing into the rectum a plug of opium half the size of a common hazlenut or filbert, to quiet the straining or constant desire to stool, or to compose the bowels, at the time the calomel pills are taken, or any time before the physician arrives; it saves time, gives repose, and has none of the ill effects of such things introduced into the stomach.

It is a great mistake that calomel is slow to operate, and that mistake consists in not knowing what its first operation is, which is to arrest the action of the bowels *within two hours*, and if enough is given it will do so, in any curable case, with the certainty almost of a specific. Some physicians hesitate, because they fear it will excite irritation—that is, aggravate the condition of things already present; they thus think, because they have seen calomel given and the symptoms soon after become worse. So have I:—first, because it is the nature of Cholera to get worse constantly—get worse every hour; and second, because so little was given, that it was simply powerless—all the injury it could effect was negative. While I am writing this, the former health officer of the port of New York during the first cholera, states that they tried every thing, and his conclusions were, that "calomel cured as often as any thing else, and if any thing was to be done it was by calomel."

While the more immediate effect of calomel in cholera, is to arrest the looseness more or less *within two hours*, then its stimulating energies begin, and at the end of six, eight, or ten hours, colored, consistent dejections appear, and then, simply with good nursing, the patient is safe, with ordinary attention.

As it is malaria, from the combination of heat, moisture and vegetable matter uniting with some unusual constituent of the atmosphere, which generates cholera; and, as this malaria is heaviest nearest the earth, persons are safer from cholera who live, or at least sleep in the upper stories of houses, as explained in my publication on Bronchitis and Kindred Diseases, eighth edition, page 317, strongly corroborated by the fact recently published, that in London, in 1848–9, epidemic cholera was fatal in the inverse proportion to the elevation of the houses above the general level—that is, from houses erected on a piece of ground forty feet higher than the general level, sixteen died of cholera out of every hundred thousand; from forty to sixty feet, eleven in every hundred thousand; from sixty to eighty, four in every hundred thousand; from eighty to one hundred, only three deaths in a hundred thousand; while in houses not over twenty feet above the general elevation, thirty-one persons died of cholera in every hundred thousand; and, without giving a special reason for it here, I only remark that temperate persons may have almost an entire immunity from cholera during an epidemic, by sleeping thirty feet or more above the ground, by eating breakfast before going out of doors in the morning; and, thirdly, by having a good fire kindled at sundown, and not going out of doors afterwards, as explained at page above quoted.

Although the whole August number was taken up with the subject of Cholera, and a great part of this number, yet I feel it important to say something towards counteracting a general and most dangerous error, disseminated and constantly repeated by newspaper editors,—very particularly so by some of the New York Daily press, and that, too, in face of the fact, that some of these papers have medical editors in their department. This fatal error is, that Cholera is a very mysterious disease, and, in the main, falls upon its victim with the suddenness and fatality of a thunderbolt. The inevitable and practical result is, that a species of terror attends an attack of Cholera, in a vast number of instances, having a more injurious effect than the disease itself. A case in hand is given in the Buffalo Republic of the 27th July:

"A strong, healthy laboring man was seized with Cholera. The moment he became aware that the disease was upon him, he grew excited, calling for all the medical aid that could be got around him. They came, administered remedies, and consulted together, and were earnest in their endeavors to do every thing in their power to save him. The man was still frantic with fear, and called upon them individually to save him. 'Save my life,' said he, 'and I will give you one thousand dollars.' His physicians tried to calm his feelings and subdue his fears, assuring him that it was absolutely necessary that he should be calm and tranquil in order to give effect to the medicine and check the disease. Fear, however, had taken such firm hold of him that he could not refrain from continued cries for help until prostrated and unable to speak, when death put an end to his sufferings and fears."

Let it be remembered by all, that *there is no positive evidence* that any man ever dies within twenty-four hours after the first onset of the disease. I make the statement with great deliberation, and certainly not without many searching inquiries and close observations. I have never yet, in a single instance, failed to find, that even *days before, something was amiss,* but so slight as not to fix attention, and almost to be unremembered in a dozen hours afterwards. I earnestly trust that educated physicians—men of age and character in the community—will make observations in this direction, and come out openly, *under their own proper signatures,* and let the people know something tangible, something practical on this death-dealing subject. How is it that in twenty years medical men have not arrived at some few general principles, practical in their nature—some few principles so intuitively truthful as to command the unanimous assent of the commonest observers. Such principles do exist, and they ought to be searched out and published by authority. For example, in the first stages of cholera in actual existence, there is *a wanting to rest;* nature, reason, common sense, instinct—all teach that rest of the most perfect kind should be observed; and yet what physician does not know how fruitlessly men fight against this inclination and perish in the contest. All classes or sects of physicians claim the successful treatment of cholera, and no doubt all are more or less successful—those who bleed and those who do not; those who give calomel and those who deprecate its employment as useless, if not fatal; those who *give* nothing but internal remedies; those who *do* nothing but make external applications; those who starve and those who feed; those who drown with water and those who deny a drop. It seems to me quite apparent that the reason all modes of treatment are more or less effectual, does not lie in the fact that cholera is not a dangerous or a critical disease, but that there must be some general principles of treatment which run through all the modes practised. If these general principles could be culled out, and, in addition, some *really first symptom* of cholera were fixed on, far earlier than the painless looseness, then not a creature need die where millions now do!

Reader of mine, in the shades of the forties, you have found more than once or twice, that in times of real difficulty, if you could not help yourself, you had to go unhelped. This is as it should be—it makes men self-reliant; he who is always helped remains a baby always, and his name and memory rot in "ninety days after"——his body. This being so, let us help ourselves, in the present dearth of help amid such myriads of doctors and certain infallible cures for cholera, and endeavor to find some two or three or more things which all "pathies" attend to in the treatment of the fearful scourge.

1st. It is becoming a matter of universal assent, that in cholera times, a painless, weakening, inodorous, watery, light-colored looseness of the bowels is actual cholera. Few die who instantly call in competent medical aid.

2d. All admit the imperative, the absolute necessity of perfect quietude from the instant the first symptom is noticed.

3d. So few deny, over their own proper names, that swallowing ice is beneficial, or, if not attainable, ice-cold water, in one or two swallows only at a time, repeated every few minutes when there is thirst, we may safely take this as a third general principle.

4th. No one denies that the looseness should be arrested without delay.

5th. That it is madness not to secure the services of a regular practising physician at the earliest moment.

6th. If at all possible, make a positive arrangement that the medical attendant shall see you once an hour, until the crisis is past.

Now, if instead of the first general principle above named, mine is substituted—that, in cholera times, the first symptom of the onset of cholera is simply a weakening uncomfortableness about the belly—then cholera will become one of the least fatal of all known diseases.

If newspaper editors were to cause these items to be universally known and believed—as the press only can do—then would I be willing that every cholera prescription ever published, except in standard medical works, should be blotted from the memory of man; and certain I am that human life thereby would be an infinite gainer.

I have now occupied some thirty pages of my Journal in giving my views on Cholera; but no subscriber will think I have given too much importance to the subject, should he be attacked himself, or have a dear child just on the verge of collapse, as the Editor had, while penning the August article on the subject, waiting until the last safe moment, in his unwillingness to give medicine, yet having an unfaltering confidence in the value of pure calomel, judiciously given, and well watched.

To sum up, then, all I have said, in a few words,

If you have, in cholera times, any reason to believe that it is attacking you, the FIRST prescription is—and it is of immeasurable importance—send for your physician; or, rather, if you happen to be from home, at your office or counting-house, get a carriage, and call on him on your way home.

2d. As soon as you enter your house, do not wait to undress, but lie down on the first bed you come to, undressing at your leisure, and let nothing pass your mouth but ice, or, if not attainable, cold water,—one or two swallows at a time, and not oftener than as many minutes apart; but if you have ice, you can eat it as voraciously as you desire,—but take neither ice nor water unless you are thirsty.

3d. This third item is conditional. If the symptoms are urgent, or you find yourself becoming nervous, and a physician cannot possibly be had within two hours,—*then* swallow ten or twenty grains of calomel, in pill, if there is sickness at stomach; if not, it will do you more good to take it on the end of a spoon-handle or case-knife, and plaster it over the back part of the tongue, washing it down with cold or iced water, taking at the same time, if so disposed, at least as much super-carbonate of Soda, as an apparent preventive, in some instances, of salivation, and wait until your physician comes.

It requires a philosopher to march up to the cannon's mouth while the match is just descending on the touch-hole, in spite of the gunner's assurance that he will not fire it off; and not less a quantum of firmness does it require to resist the incessant importunities of those we love, to be doing something; if you have any disposition to gratify them, without injuring yourself, and yet do some additional good, introduce into the rectum a long piece of opium, which, in the shape of a ball, was half as large as a common-sized filbert, or, as called by others, hazlenut.

"Do let me alone," is the very frequent petition of a cholera patient, unless he is a stranger and has no money; in that case, there is no kind of necessity for a repetition of the prayer.

Since the first four pages of this September article on Cholera were put in type, I have purchased the August number of the New York Medical Gazette, the regular exchange not having come to hand; and having read it since its first publication, I did not wish to be without it—and such, I hope, will be the feeling of the subscribers to the Journal of Health for years to come—for somehow or other, any man who takes and pays regularly for a periodical, gets to like it and the editor too; or, at the very least, to feel out of sorts if he does not get it at the appointed time. The Gazette says of our August No., as an offset to its commendation, that it regards,

1st, The definition of Cholera as defective.

2d, The theory radically inadequate.

3d, The treatment imperfect.

This criticism is correct in the main; for as to the definition, designing it for popular use, we wanted to present one main, easily understood, and easily remembered idea. I did precisely as I have a thousand times wished our ministers would do, that is, to give in each sermon one clear and grand idea, impressed in such a manner, that on his way home, the hearer is not inclined to talk or think of anything else. Time nor the daily battle with the world will ever burn that idea out. If clergymen would do this, they would not run out of ideas in every five or six years, and resign on account of ill health. I name this as an incidental preventive of Cholera; for it is enough to cause more than cholera to be in the chase of new ideas in mid-summer, for weeks at a time, and yet not a single one be caught—not in a whole year. Whose health wouldn't give out under such circumstances? The one-idea sermon has two great advantages—it would be necessarily short, and being to the point, too, there would not be a sleepy or "forgetful hearer of the word" in all the congregation. So in my definition of Cholera, I wanted the unprofessional reader to see, and feel, and remember the one main, practical idea, that Cholera was excessive motion of the bowels, and that its cure, except in advanced stages, was perfect quietude.

2d. "Theory inadequate." I often think myself that theory is a fool, and theorizers foolees. But whatever may be the respective merits of my theory, and that of the Gazette, both lead to the same practice; for in answer to the question, "What shall we do in Cholera?" proposed by many city friends, subscribers, and former pupils, the Gazette advises four things: 1st, a physician; 2d, laudanum; 3d, ice; 4th, "*all previous treatment being palliative*," calomel in quantity proportioned to the violence of the attack, taken by being plastered on the tongue and washed down with ice-water. Now, if the Editor of the Medical Gazette had not have been old enough to be our greaty-great-grandfather, and forgotten, perhaps, more than we ever knew about general medicine, we might have concluded that the advice he gave in his August number, issued August 1st, was taken from the August number of the Journal mailed to exchanges, 20th July.

3d. "Treatment imperfect." And so it was purposely designed. I wished the patient to know no more than what it was necessary to do while his physician was coming; and although, as the Gazette admits, "in very many cases there could be no better practice," and nothing more would be needed, there are some cases which require more energetic means than ten or fifteen grains of calomel. My object was not to cause the patient to feel that he was fully armed at all points; for then he would not send for a physician at all; and one of the main objects of the article would have been wholly frustrated, that is, the early call of the family physician, which the editor himself insists upon, is the very first and most important thing to be done in every instance. I think one of the best points in the August number is the scantiness of the advice in reference to the actual medical treatment. It is not my intention that this Journal shall ever contain an article that, by any torture, can be made to take the administration of medicine out of the hands of the regularly educated and honorable allopathic practitioner, except in cases where the delay of an hour or two would be death. I do not say that I will even do this, except in very rare cases, which, indeed, I might do in justice to those of my subscribers who reside in the country, and may not be, as many are, within ten miles of a physician.

I should have been glad, and the public would have been instructed, if the Editor of the New York Medical Gazette had given his opinion as to the truth of the main idea of my Cholera article, to wit: that, in cholera times, any "weakening, abdominal uncomfortableness" should be regarded as the forerunner of actual cholera, and that, at that point, quietude is a prompt, perfect, and permanent cure. Dr. Rees is a veteran in the Medical Profession, an author of celebrity, and of large and long opportunities of observation,—and these, combined with a classical education, entitle his opinions (as they really receive) to the respectful consideration of educated practitioners, and he, and Dr. Mott, and Horace Green, and Mussy, and Warren, and Jackson of Philadelphia, are the very men who ought to have come forward long ago and popularized the nature, first symptoms, and the un-medical treatment, while waiting for the physician's arrival. The public has honored and enriched these men, and had a right to look to them when the scourge came; but, as far as I know, they have kept in the shade, while younger men have been afraid; and thus, without a light or a guide, the people have died grasping at straws, which anonymous scribblers and ignorant or unprincipled vendors of cholera preventives and cholera specifics have thrown in their way.

Another last word as to the value of calomel, *alone*, in cholera. Taking allopathic practice as our guide, may we not cull out a *seventh* first principle in the management of Cholera, as follows: Very few, indeed, of regular practitioners ever attempt the treatment of a single case of cholera without the use of calomel, or of mercury in some other form; some combine opium, others use calomel alone—both are unquestionably successful. Cannot the unprejudiced general reader see, then, that after all, calomel is the efficient agent,—and, inasmuch as opium undeniably produces fatal effects, sometimes in the form of convulsions, congestions and water on the brain, while by detaining the calomel in the system too long, it causes salivation, mercurial fever, loosening the teeth, eating away the gums, and sometimes large holes in the cheeks of children, which nothing but death can arrest,—I ask the simple question, is it not imprudent, to say the least of it, to advise any one not a physician to take opium in any form, or opium in combination with calomel, for cholera, or anything else, unless the physician is by to superintend its administration? What I glory in, as a medical practitioner, is to be on the safe side—my motto, from earliest practice, has been, rather let a patient die without medicine, than with too much.

I know of no paper published on the subject of Cholera, which has been so largely and so generally copied from, as that of our August Number. Physicians from different parts of the country have applied for it. The secular newspapers have, as far as I have seen, given it a unanimous and friendly commendation; while the Medical press has also regarded it with favor, one of them declaring, that as a general rule, "there could be no better practice," and that "it is greatly to be preferred to any newspaper article" that has come under its notice. To my medical brethren I desire to say, that they will be disappointed in it. It was not designed to instruct them, but to present to the people for practical observance, some general, main principles, intuitively seen, readily understood, and easy to be remembered. Medical men entertain different views as to the theory of the disease,—but that is pretty much like the "*how*" of the origin of a fire; the fire is there, and all agree that water must be applied to put it out. So all classes of physicians admit that the "looseness" must be speedily arrested; and the main reliance of legitimate medicine is calomel and its combinations. Where I stand out from them, is in the manner of using the calomel. Now, there is something so curious in this, that I wish to draw editorial attention to the subject; for it must be admitted, that a new profession has arisen among men, and that the Press vies with the Pulpit in the regulation of the world; reforms cannot progress without its aid—prejudices cannot be annihilated, and newer and more truthful views substituted, without its co-operation. Christian men, especially, ought to understand that a united tripod will sweep before it the Faculty, the Pulpit, and the Bar, as the whirlwind sweeps the chaff of the threshing-floor; and the time has already come when young men should be educated for the sanctum with as much directness as they are educated for law, physic, or divinity. It used to be said, with resistless truth, "like people, like priest;" and not less so is it to-day, *as the papers*, so are the people. For example, look at German newspapers—look at German principles in the United States,—infidel in sentiment, they openly propose in practice the abolition of the Sabbath, the marriage tie, and, in effect, all commercial municipal law. But what has this to do with Cholera? Much, every way. I want the Press to understand its position, its power, and its duty,—and, feeling its high responsibility, lend me a hand in ameliorating human suffering, by widely diffusing correct and consistent views as to the nature of a disease, which, since its malignant appearance at Jeddore, in eighteen hundred and seventeen, is estimated to have destroyed about eighteen millions of the human family. Let the press, then, join in diffusing knowledge among men, as to four great points: THE NATURE, THE CAUSES, THE PREVENTION, THE EARLY TREATMENT of Epidemic Cholera.

*Its* NATURE, *a weakening condition of the bowels.*

*Its* CAUSES, *dirt and intemperance, in eating, quite as much as in drinking.*

*Its* PREVENTION, *cleanliness, temperance, and a quiet mind.*

*Its* EARLY TREATMENT, *quietude, and the prompt call of a physician.*

I believe that on these four points there is a perfect unanimity among all classes of physicians, everywhere; but the people, the masses, somehow or other, do not *feel its truth*, and that is because they have not been informed with a precision and consistency sufficient to arrest the attention and secure the assent of the understanding.

Another reason for the digression made awhile ago, is, I wished the attention of editors drawn to the fact, that while a proper self-respect and common policy should prompt them to leave purely medical questions to be discussed by medical men, yet there are some points, of a practical character, upon which they may very properly exercise a dignified and judicious observation, and one of these points is the administration of calomel in cholera.

If I were attacked with undisputed cholera, I would do four things:

1st, Lie down; 2d, eat ice, if thirsty; 3d, bind a piece of woollen flannel tightly around the abdomen; 4th, take calomel.

This fourth item requires a more extended mention. I would take an amount supposed to be sufficient. If it did not arrest the passages within two hours, I would *double* that amount, and continue to *double each last dose* at the end of each second hour, until the disease was arrested.

Now it is the reason for this, to which I wish to direct editorial attention, as entirely competent to decide whether the practice is wise or not.

Since calomel, or calomel with opium are given as a standard prescription in allopathic practice, and both with success, it seems plain that calomel is the efficient agent.

Dr. Jackson, who, for a long period, was in the service of the Hon. East India Company, says, that pure calomel was "a leading, indispensable remedy in the treatment of malignant cholera, *none other being thought of in India*," where the cholera has raged with all its terrible malignity for more than thirty-five years.

Why, then, do some physicians in this country combine with the calomel some form of opium? To "anchor it," they express themselves; to hold it in the system; to keep it from passing off without accomplishing anything. The argument is this: a small force held on, against a larger force at once applied. Fire makes water boil—a greater fire makes it boiler.

The East India practice, where cholera is seen in a more furiously malignant form than can be witnessed here, is to increase the force of the agent—that is, give larger doses; and if near forty years' experience, in the most violent forms of the disease, has led to the general adoption of the practice, in the most enlightend part of India,—that is, under the more immediate eye of the East India Company,—the fair presumption is, that being "*the*" practice in severer forms, it is the better practice in milder cases.

But why do not physicians here increase the force—that is, the quantity of calomel? They are afraid. I do not mean to say of my brethren, that they are afraid of popular prejudice, or of pecuniary loss by abatement of practice,—because the true physician knows no mortal fear; it is the fear of humanity, that he may injure his fellow-citizen, his neighbor, his friend, who has placed his life in his hands—higher confidence than this, can no man place on earth. But what is he afraid of? The baseless fabric of a vision.

The ground of this fear is, that by a few grains of calomel, comparatively speaking, consequences severely injurious have sometimes taken place—effects which last for life; reasoning, that if a small amount of gunpowder occasions disastrous results when fire is applied, a greater amount of powder would be attended with proportional injury. Reasoning by comparison is always dangerous. A gentleman, reading the August No., concluded he would carry a few ten-grain calomel pills in his pocket, and applied to a German apothecary to put up half-a-dozen for him. "What are you going to do with ten-grain calomel pills?" in evident astonishment. "I will swallow them, if necessary." "Are you going to kill yourself?" And when it is remembered that German apothecaries are scientific men, educated expressly for the purpose, the reader may see the extent of the general prejudice when it pervades the intelligent classes.

Will any physician in New York, or out of it, who opposes ten, twenty, fifty-grain pure calomel doses, inform me by mail, at my expense, if he ever knew a man to take a hundred grains of calomel at a time; if not, then all that he imagines as to large doses of calomel being injurious, is purely hypothetical.

Calomel in a man is, in some respects, like sugar in a cup of coffee: you can sweeten the coffee to a certain point—beyond that you cannot go; the coffee takes up no more, and the sugar falls to the bottom, and no use is made of it. In a state of disease, the human system will take up a certain required amount of a single dose of calomel, and will take up no more; the remainder is hurtless and useless, and passes from the system mainly unchanged. This was the principle adopted by John Estin Cook, our honored preceptor, who had, in our opinion, one of the greatest purely medical minds of this or any other age or nation: but he was considered, on the subject of calomel, as mad as a March hare, or as the Apostle Paul, and for the same reasons, that is Paul, not the hare:

1st. He was fifty years ahead of his time.

2d. He, like most minds of mark, was not understood. The fog of prejudice was so thick, that his express declarations would be interpreted to the very reverse of his intentions. The impression became so general, that he "*gave so much calomel*," he was scarcely able to make a living by the practice of his profession. The same is said of the immortal Harvey. The actual facts were, that in any given case, he would, in the course of his treatment of it, give *less* calomel than other physicians. "Young gentlemen," he would say, with his manuscript lecture in one hand, and his spectacles astride the fore-finger of the other, sawing the air with great earnestness, "the difference between us is this: I give a man a single dose of calomel—you call it a large one—and I cure him up in a day or two; you give a little at a time, often repeated, and at the end of many days he is convalescing,—you, in the mean time, having given in the aggregate five times as much as I would."

In general practice, he did not often give more than five or six grains at a time; but in urgent cases, where danger was imminent, he was a perfect Napoleon—he feared nothing when his patient's safety was involved—and I have known him to give from one hundred to three hundred grains of pure calomel at a single time, with the most triumphant success, in the restoration of the patient to perfect health, without salivation or any appreciable subsequent ill result. It is known, too, that Southern physicians, thrown as they often are by frequent and great exposures, into desperate situations, have been known to grope their way at midnight to the calomel jar in their offices, and catch it up in their fingers, as men do flour from a barrel, and swallow it down, and be visiting their patients within the next twenty-four hours. If the reader will turn to one of the old dispensatories, he will find that five grains of the sub-nitrate of Bismuth was considered a dose which *might be!* increased gradually to twelve or fifteen grains at a time; and it was considered dangerous, because poisonous, to go much beyond that. I use it in certain forms of loose bowels, in doses of a teaspoonful, or a hundred grains, three times a day, and that with admirable advantage, apparently *without any medicinal effect whatever*, seeming to do good by acting as a mechanical coating over the tender surface of the intestines. And yet for generations it had been dribbled out in doses of five and ten grains,—the tyrant AUTHORITY wielding, as it always does, the sceptre of a despot. Here is a case parallel with that of calomel. Men have drawn back with consternation at *large doses*, without ever having had the courage to take or give a large dose, and see for themselves what its effects would be, basing their practice on mere conjecture from the effects of small doses, or in combination with other remedies.

In an able historical article in the New York Herald of the 2d August, the writer says that he "was, at one time, in 1834, attacked in a most violent manner with Asiatic Cholera, when he took about six or seven even teaspoonsful of calomel before one remained on his stomach. Reaction then commenced, and he was next day enabled to walk out. The only external remedy used was the temporary application of a mustard plaster over the stomach. The only inconvenience he felt was a slight ptyalism, from his susceptibility to the influence of mercury. But this was nothing to dying. He then tried the same treatment in other violent cases with the most uniform and perfect success. In 1840 he experienced another attack of cholera in Liverpool, and again cured himself by similar treatment. He became acquainted with Dr. Jackson, who had enjoyed great experience in the treatment of the disease during a long period in the Hon. East India Company's service. He informed us that the calomel practice, in the form and manner we have described it, formed the most successful practice of any other."

While such are my sentiments as to giving calomel, largely, in desperate cases, I do not advocate its free use in general practice, where I have seldom given over four grains at a time, and not oftener than once a week; and with certain nauseants not necessary to be named in a popular Journal, I find that it does not fail once in a thousand times to act within the twelve hours, and hence nothing is given afterwards to carry it off, as it takes care of itself. It is the weak-minded admirer of a great theorist who runs the principle into the ground, making the step from the sublime to the ridiculous so short, that the prejudiced and the hide-bound "have it all their own way."

Gentlemen of the Press, having taken a common-sense view of the statements I have made, do you feel prepared to abide by the pure calomel treatment, administered with a bold hand, in case you are seriously attacked yourselves? Then let me arm you with a succinct statement of the advantages of it.

1st. Calomel is tasteless, and therefore can be easily taken by small babies and grown ones.

2d. It will remain on the stomach when even water is ejected with a powerful force the moment it is swallowed. Can't you see the utter inutility of every other remedy, of even a specific that would cure every case in ten minutes after it was swallowed, when you can't keep it in the stomach a half minute?

3d. Calomel costs almost nothing, is to be had at every drug store, and is furnished without charge at the dispensaries. What is the use of talking about the advantages of pure brandy to the multitudinous poor, who seldom have a shilling ahead? Then again, where is that brandy? Besides, every physician knows it will kill any man who relies upon it in any case of actual Cholera.

4th. A double or tenfold dose of calomel can't kill you. Death, simply by an overdose of calomel, is impracticable. But if you take an overdose of opium, in any of its forms, alone or with calomel, or with any other medicine, a very speedy death is certain; while in a quantity not considered a *very large dose*, it very frequently, when given for loose bowels in children, gives water on the brain,—and, in adults, causes convulsions, congestion, typhoid fevers, and death—death, too, in one of its worst forms,—allowing you to linger for hours and days in an unconscious stupor, and in that state to pass from all we love. Let not such a death be mine; let my eyes be open, and my intellect as clear as the dewdrop of the morning, when that great hour comes to me.

Trusting that what I have said will invite the unprofessional reader to reflection, to think for himself, and that medical men may be stimulated to renew their investigations, with a view to more truthful and more practicable ideas on a subject which involves the lives of unborn millions, I here introduce two or three articles from other sources, not endorsing what is said of anodynes, stimulants, or the infinitessimal dilutions,—the last being as yet a *terra incognita*, an unexplored country, a domain where I would like to travel, had I the time which thousands have so much of, yet do not use, except in studying how to kill it often. What a murder—what a profanation. I am inclined to think there is something in Homœopathy; for, as far as my observations have gone, it acts on the principle of the bread-pills of the regulars—they give their bread-pills with a serious face and a confident anticipation of good results; and I see no reason why the little white ones should not do as well—they certainly go down easier.

